# Post-transplant MASLD: a risk-stratified approach to assessment and personalized management

**DOI:** 10.3389/fmed.2026.1878245

**Published:** 2026-06-10

**Authors:** He-Yu Huang, Zhong-Qi Fan, Guo-Yue Lv

**Affiliations:** 1 Department of Hepatobiliary and Pancreatic Surgery, General Surgery Center, First Hospital of Jilin University, Changchun, Jilin, China; 2 China-Singapore Belt and Road Joint Laboratory on Liver Disease Research, First Hospital of Jilin University, Changchun, Jilin, China

**Keywords:** liver transplantation, metabolic dysfunction-associated steatotic liver disease, non-invasive assessment, personalized management, risk stratification

## Abstract

Metabolic dysfunction-associated steatotic liver disease (MASLD) has become a major indication for liver transplantation (LT) and is expected to account for an increasing proportion of transplant candidates worldwide. As long-term post-transplant survival continues to improve, post-LT MASLD has increasingly emerged as a major metabolic complication affecting long-term prognosis. Unlike MASLD in the non-transplant setting, post-LT MASLD is strongly influenced by transplant-specific factors, including recipient metabolic dysfunction, immunosuppressant use, graft-related characteristics, and metabolic alterations following transplantation, resulting in substantial heterogeneity in clinical presentation and disease progression. Current evidence suggests that recipient-related metabolic factors, particularly obesity, type 2 diabetes, dyslipidemia, hypertension and post-transplant weight gain, play central roles in the pathogenesis, whereas donor-related characteristics may contribute predominantly to early liver fat accumulation. Importantly, the clinical significance of post-LT MASLD extends beyond simple steatosis and is closely associated with fibrosis progression, cardiovascular events, renal dysfunction, and other systemic metabolic complications. Nevertheless, the current literature remains highly heterogeneous. This variability likely reflects evolving disease definitions, the frequent failure to distinguish recurrent from *de novo* MASLD, and inconsistencies in diagnostic modalities across studies. Moreover, assessment models originally developed for MASLD in the general population may not be fully applicable to LT recipients. In this review, we comprehensively summarize the epidemiology, phenotypic heterogeneity, risk factors, pathogenesis, diagnostic and surveillance strategies, and emerging individualized management approaches for post-LT MASLD, with particular emphasis on a long-term management framework based on risk stratification. Accordingly, future management may need to focus less on steatosis alone and more on identifying patients at high risk for progressive hepatic and extrahepatic complications. A more comprehensive assessment integrating metabolic, hepatic, cardiovascular, and transplant-related factors may improve individualized surveillance and therapeutic decision-making. Advances in non-invasive imaging, multidisciplinary management, and artificial intelligence-based prediction models may further support individualized and risk-oriented care in post-LT MASLD.

## 1 Introduction

Metabolic dysfunction-associated steatotic liver disease (MASLD) has emerged as one of the most prevalent chronic liver diseases worldwide, affecting more than 30% of the global population, and is increasingly recognized as an important cause of cirrhosis and hepatocellular carcinoma (1, 2). Parallel to the global rise in metabolic syndrome, the number of patients with MASLD-related end-stage liver disease has markedly increased, establishing MASLD as one of the leading indications for liver transplantation (LT) (3–5).

Importantly, MASLD after LT includes both recurrent and newly developed (*de novo*) steatotic liver disease (SLD), each of which is frequently observed and appears to be increasing following transplantation (6). Although short-term post-transplant outcomes are generally favorable (6), persistent metabolic dysfunction after LT is associated with a higher risk of cardiovascular events, liver-related complications, and adverse long-term outcomes, thereby posing a major challenge for long-term post-transplant management (7–9).

Importantly, post-LT MASLD should be considered a distinct metabolic condition rather than simply a continuation of disease in non-transplant populations, owing to its unique transplant-related characteristics (6, 10). The interplay among recipient metabolic dysfunction, immunosuppressive therapy, donor- and graft-related factors, and metabolic alterations after transplantation contributes to substantial clinical heterogeneity.

Nevertheless, current management strategies are largely extrapolated from studies conducted in the general MASLD population, while specific evaluation frameworks tailored to LT recipients remain insufficiently developed. Reliance on isolated clinical indicators may not adequately capture overall disease risk, thereby limiting early identification and intervention in high-risk patients (11, 12). Accordingly, the development of multidimensional risk stratification models integrating metabolic, hepatic, cardiovascular, and transplant-related parameters is essential for optimizing long-term management.

In this context, a critical unresolved question is whether post-LT MASLD can continue to be managed using conventional paradigms or instead requires novel risk stratification strategies that account for transplant-specific factors. This review therefore aims to systematically discuss the pathogenesis, risk assessment, and tailored management strategies for post-LT MASLD, with particular emphasis on early surveillance, optimized intervention, and individualized management approaches, in order to inform future clinical practice (11, 12).

## 2 Evolving landscape and phenotypes of post-transplant MASLD

### 2.1 Recurrent and *De Novo* MASLD

Post-transplant MASLD can be classified into recurrent and *de novo* ones. Recurrent MASLD refers to the persistence or recurrence of pre-existing metabolic abnormalities after transplantation, whereas *de novo* MASLD develops in recipients whose primary liver disease was unrelated to metabolic liver disease (13–15). Notably, no standardized time point has been established for defining *de novo* MASLD after liver transplantation; it can occur at any time after transplantation, with approximately two-thirds of cases developing within the first year post-transplant (13, 16). Although these entities are frequently considered together in clinical practice and research, their underlying mechanisms and clinical characteristics may differ. Recurrent MASLD is more commonly associated with the persistence of pre-transplant metabolic abnormalities, whereas *de novo* MASLD appears to be more closely related to post-transplant metabolic alterations and immunosuppressive therapy-related metabolic disturbances (15, 17). Recurrent MASLD is generally associated with persistence of pre-transplant metabolic dysfunction, including obesity, type 2 diabetes mellitus, and metabolic syndrome, whereas *de novo* MASLD appears to be more closely related to post-transplant metabolic alterations, immunosuppressive therapy, and weight gain after LT (13, 15). Recurrent MASLD may also demonstrate earlier steatosis recurrence and fibrosis progression, likely reflecting persistent systemic metabolic dysfunction (13, 16). In contrast, *de novo* MASLD may show a more heterogeneous clinical course influenced by immunosuppressive exposure and post-transplant metabolic changes (15). However, direct comparisons of long-term outcomes between these phenotypes remain limited. Nevertheless, current studies have not consistently distinguished between recurrent and *de novo* MASLD, and available outcome data remain heterogeneous. This insufficient distinction between the two entities has, to some extent, limited accurate characterization of their natural history and clinical outcomes (3, 13–15).

### 2.2 Epidemiological trends of post-transplant MASLD

Registry studies from multiple countries have identified MASH and MASH-related end-stage liver disease as among the fastest-growing indications for liver transplantation (LT) (3, 4, 18). This epidemiological shift has not only altered the baseline characteristics of transplant recipients but has also resulted in persistent exposure to metabolic risk factors after transplantation, thereby creating a favorable setting for the development of post-LT MASLD.

Against this background, increasing attention has been directed toward the epidemiology of post-LT MASLD. Overall, current evidence consistently indicates a high prevalence of post-transplant steatotic liver disease (SLD), with rates increasing over time after transplantation (6, 7, 16, 19–21). Nevertheless, substantial variability exists across studies, likely reflecting differences in study design, ethnicities, diagnostic approaches (including liver biopsy and imaging-based assessment), and disease definitions. Importantly, the transition in nomenclature from nonalcoholic fatty liver disease (NAFLD) to MASLD has introduced additional heterogeneity in diagnostic criteria across studies. To date, most available data remain based on the NAFLD framework, whereas prospective studies applying MASLD-specific definitions are still relatively limited (6, 7, 16, 20, 21).

A meta-analysis including multiple studies reported a pooled prevalence of post-LT steatosis of approximately 39%, although marked interstudy variability was observed, with reported prevalence ranging from 12% to 88% (6). Further analyses suggested substantial geographic variation, with relatively higher prevalence rates observed in the United States and Latin America, compared with lower rates in Europe and Asia (6). Similar trends have also been reported in other meta-analyses and in studies using vibration-controlled transient elastography (VCTE) or metabolic dysfunction-associated fatty liver disease (MAFLD)-based diagnostic criteria (7, 16, 20, 21). In addition, a prospective study from India reported a steatosis prevalence of approximately 33% in living-donor recipients (22).

From a longitudinal perspective, the incidence of post-transplant SLD appears to increase progressively over time, with an estimated rise of approximately 11% per decade after transplantation (6). Moreover, recurrent steatotic liver disease appears to be more common than *de novo* disease, with the risk of recurrence reported to be up to five-fold higher than that of *de novo* disease (6, 13, 16, 23). Collectively, these findings suggest that pre-existing metabolic dysfunction continues to exert an important influence after transplantation and may contribute differently across distinct disease phenotypes.

### 2.3 Phenotypic heterogeneity and risk stratification

Post-LT MASLD is characterized by substantial phenotypic heterogeneity. This heterogeneity is reflected not only in the distinction between recurrent and *de novo* disease, but also in varying combinations of metabolic risk burden, alcohol exposure, and multisystem complications (17, 19). From a risk stratification perspective, these phenotypic differences can be understood as the result of multidimensional interactions.

First, patients differ markedly in metabolic burden and systemic complications, underscoring the heterogeneity of post-LT MASLD (21). Second, exposure-related factors also contribute to phenotype formation. With the evolution of steatotic liver disease nomenclature, recognition of the MetALD overlap phenotype in LT recipients has become clinically relevant, highlighting potential interactions between metabolic dysfunction and alcohol exposure (24–26). In addition, as a systemic metabolic disorder, MASLD is frequently accompanied by cardiovascular, renal, and endocrine abnormalities. This multisystem involvement not only reflects the complexity of disease phenotypes but is also closely associated with long-term outcomes after transplantation (17, 27).

Accordingly, post-LT MASLD phenotypes may be viewed as the combined result of metabolic burden, exposure-related factors, and systemic complications, with implications for individualized follow-up and management.

## 3 Risk factors and clinical outcomes

### 3.1 Risk factors

First of all, recipient-related factors appear to play a dominant role in disease initiation and progression, whereas donor- and transplant-related factors may exert synergistic or amplifying effects at different stages of disease evolution (6, 28).

Among recipient-related factors, metabolic dysfunction represents the principal driving mechanism. Obesity, type 2 diabetes mellitus, dyslipidemia, hypertension, and metabolic syndrome have all been closely associated with post-transplant steatosis and disease progression (6, 21, 22). In addition, the underlying etiology of liver disease may influence post-transplant risk. Recipients with prior MASLD or alcohol-related liver disease are more likely to develop SLD, and these conditions may exert additive effects within a shared metabolic background (6, 25).

Emerging evidence suggests that genetic variants in both donors and recipients may influence disease development as some established MASLD-associated variants, including PNPLA3 rs738409 and TM6SF2 rs58542926, were associated with post-transplant SLD (29, 30). In addition, certain single-nucleotide polymorphisms (SNPs), such as HSD17B13 rs72613567, may exert protective effects and participate in disease risk modulation, although their clinical significance remains to be further clarified (31).

Additionally, donor-related factors appear to limitedly affect the development of post-transplant hepatic steatosis. Systematic reviews and meta-analyses have suggested that donor-related characteristics are not the principal determinants of post-LT steatosis, and their impact on sustained disease progression and long-term outcomes remains incompletely defined (6). By comparison, transplant-related factors may amplify effects on disease development. Immunosuppressive therapy, particularly calcineurin inhibitors and corticosteroids, may promote hepatic steatosis through the induction of post-transplant metabolic disturbances, including diabetes mellitus, dyslipidemia, and hypertension (23, 27). In addition, post-transplant weight gain, obesity, and dyslipidemia are common after LT and may further increase metabolic burden, thereby contributing to the development of post-transplant MASLD (23, 32). Beyond these metabolic factors, surgical injury may also participate in the pathogenesis of post-LT MASLD. Ischemia–reperfusion injury (IRI) can induce oxidative stress, inflammatory responses, and mitochondrial dysfunction, thereby disrupting hepatic metabolic homeostasis and aggravating graft injury (23). Notably, steatotic grafts themselves appear to be more susceptible to IRI, suggesting a potential bidirectional interaction between these processes (23).

Collectively, post-LT MASLD is driven by both metabolic dysfunction and transplant-related factors. Integrated risk assessment may help identify high-risk patients and support individualized management strategies (Table 1).

**Table 1:** Risk factors for post-transplant MASLD.

Domain	Specific factors	Underlying mechanism	Main clinical impact	Evidence and notes
Recipient-related (core drivers)	Obesity, type 2 diabetes, dyslipidemia, hypertension	Insulin resistance and metabolic dysregulation	Steatosis, MASH, disease progression	High; primary driving factors (6, 21, 22)
	Primary liver disease (MASLD, ALD)	Persistent metabolic milieu	Recurrent or *de novo* MASLD	High; additive effect with metabolic factors (6, 25)
	Genetic susceptibility (e.g., PNPLA3 rs738409 and TM6SF2 rs58542926)	Altered lipid metabolism and genetic predisposition	Increased susceptibility to steatosis and progression	Moderate; genetic modifiers with potential additive effects (29, 30)
	Protective variants (e.g., HSD17B13, rs72613567)	Reduced oxidative stress and lipid-mediated injury	Lower risk of recurrent MASLD	Moderate; emerging evidence (31)
Donor-related	Donor steatosis	Increased baseline lipid burden	Early graft steatosis	Moderate; limited impact on long-term outcomes (6)
Transplant-related (amplifiers)	Immunosuppressive therapy (CNIs, corticosteroids)	Induction of diabetes, dyslipidemia, hypertension	Increased metabolic burden and steatosis risk	High; key amplifying factor (23, 27)
	Post-transplant weight gain, obesity, dyslipidemia	Increased lipid accumulation	Steatosis and MASLD development	High; modifiable factor (23, 32)
	Ischemia–reperfusion injury (IRI)	Oxidative stress, inflammation, mitochondrial dysfunction	Graft injury and metabolic disturbance	Low–moderate; indirect evidence with mechanistic support (23)

ALD, alcohol-related liver disease; CNI, calcineurin inhibitors; IRI, ischemia–reperfusion injury; IR, insulin resistance; MASH, metabolic dysfunction-associated steatohepatitis; MASLD, metabolic dysfunction-associated steatotic liver disease; NAFLD, non-alcoholic fatty liver disease; SNPs, single-nucleotide polymorphisms.

### 3.2 Clinical outcomes and prognosis

Despite the high prevalence of post-LT MASLD, its impact on long-term clinical outcomes remains controversial. On one hand, several studies have suggested associations between post-LT MASLD and graft dysfunction, fibrosis progression, and increased mortality risk (33, 34). On the other hand, multiple studies did not demonstrated a significant effect on overall patient survival or graft survival (6, 15, 35, 36). Data regarding the prognostic impact of MASH remain inconsistent. Some studies have reported that *de novo* MASH may be associated with increased mortality, whereas others have not identified significant differences in clinical outcomes (8, 14, 35).

These inconsistencies likely reflect multiple contributing factors. First, most studies have not clearly distinguished recurrent from *de novo* MASLD, thereby overlooking potential biological differences between phenotypes. Second, heterogeneity in diagnostic criteria and the evolving definitions of steatotic liver disease have further complicated comparisons across studies. In addition, differences in immunosuppressive regimens may influence metabolic status and thereby confound outcome assessment (6). Beyond liver-related outcomes, MASLD has also been associated with a broad range of extrahepatic adverse events. Cardiovascular risk is substantially increased and has become one of the leading causes of mortality in this population (33, 34). Furthermore, metabolic complications and renal dysfunction may also adversely affect long-term prognosis.

## 4 Pathogenesis

The pathogenesis of post-LT MASLD has not yet been fully elucidated and is currently considered to represent a complex multifactorial process driven by genetic susceptibility, metabolic dysfunction, and transplant-related factors (37). Compared with MASLD in the non-transplant setting, LT recipients are exposed not only to persistent pre-existing metabolic risk factors but also to long-term immunosuppressive therapy and post-transplant metabolic alterations, thereby increasing susceptibility to hepatic steatosis and disease progression.

### 4.1 Metabolic dysfunction and transplant-specific factors

Insulin resistance (IR) is widely regarded as a central mechanism underlying the development of post-LT MASLD. IR promotes intrahepatic lipid accumulation through increased fatty acid influx, enhanced *de novo* lipogenesis, impaired fatty acid β-oxidation, and reduced lipid export (12). Persistent disturbances in glucose and lipid metabolism may further induce oxidative stress, endoplasmic reticulum stress, and chronic low-grade inflammation, thereby driving the progression from simple steatosis to MASH and fibrosis (37).

In the setting of LT, these metabolic abnormalities may be further amplified by transplant-specific factors. Immunosuppressive therapy is considered an important contributing factor, as corticosteroids, calcineurin inhibitors, and mammalian target of rapamycin (mTOR) inhibitors may all affect glucose homeostasis, lipid metabolism, and body weight regulation, thereby promoting hepatic steatosis and disease progression (38). In addition, perioperative stress and inflammatory responses may impair pancreatic β-cell function and insulin sensitivity, further disrupting post-transplant metabolic homeostasis (39).

### 4.2 Gut–liver axis, inflammation, and fibrosis progression

Beyond metabolic dysfunction, disruption of the gut–liver axis and persistent inflammatory responses also contribute to disease progression. Gut microbiota dysbiosis and increased intestinal permeability may facilitate the translocation of endotoxins into the portal circulation, thereby activating intrahepatic inflammatory signaling pathways. In parallel, farnesoid X receptor (FXR)-related disturbances in bile acid metabolism may also participate in hepatic lipid accumulation and inflammatory amplification (40).

Persistent lipotoxicity, oxidative stress, and chronic inflammation may further activate Kupffer cells and hepatic stellate cells, promoting extracellular matrix deposition and fibrosis development. In LT recipients, ongoing metabolic dysfunction and altered immune status may further amplify these processes, thereby accelerating disease progression.

## 5 Diagnosis and monitoring

The diagnosis and monitoring of post-LT MASLD require both accurate disease identification and longitudinal risk assessment. Unlike patients with MASLD in the non-transplant setting, LT recipients exhibit distinct hematologic profiles, immune status, and metabolic backgrounds, which may limit the applicability of conventional diagnostic tools (6, 12).

### 5.1 Liver biopsy: gold standard and its limitations

At present, liver histology remains the gold standard for the diagnosis of SLD, allowing not only accurate detection of hepatic steatosis but also confirmation of MASH and assessment of fibrosis stage (41, 42). However, liver biopsy is difficult to implement as a routine tool for long-term dynamic surveillance. Consequently, in the management of post-LT MASLD, liver biopsy is more appropriately reserved for decision-guiding evaluation in selected clinical scenarios rather than for universal screening.

**Figure 1:**
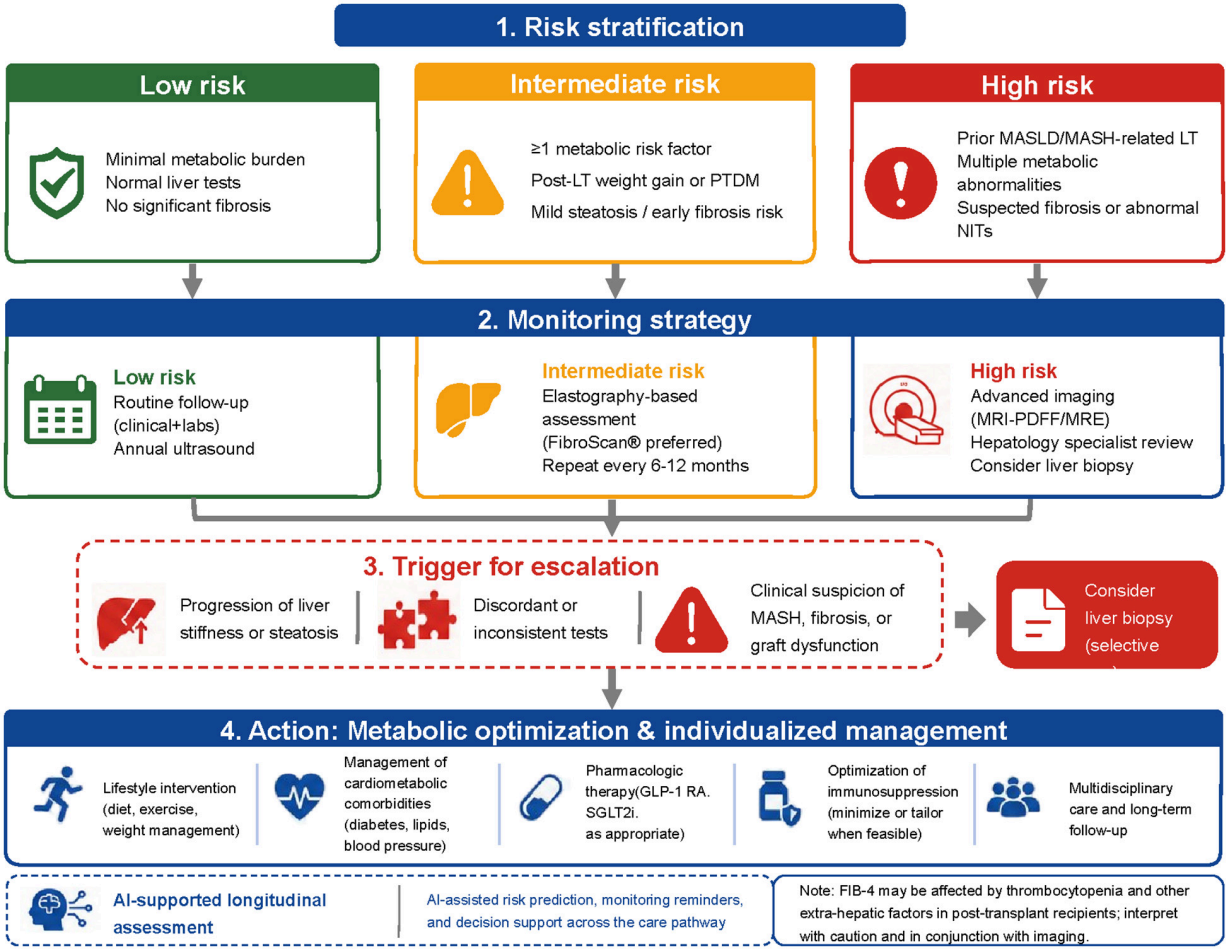
Risk-stratified monitoring and management pathway for Post-LT MASLD. The proposed framework consists of four sequential components: (1) Risk stratification, identifying patients according to overall metabolic and fibrosis risk; (2) Monitoring strategy, applying longitudinal non-invasive surveillance based on individual risk profile; (3) Trigger for escalation, recognizing clinical or non-invasive indicators of disease progression; and (4) Action: metabolic optimization and individualized management. Low-risk patients generally exhibit stable metabolic status without evidence of significant fibrosis, whereas high-risk patients may present with progressive fibrosis, multiple metabolic complications, or suspected graft dysfunction. Surveillance intensity may vary according to risk level. Potential triggers for escalation include worsening metabolic abnormalities, progressive liver stiffness, increasing steatosis burden, discordant non-invasive test results, or suspected graft dysfunction. Management may involve a multidisciplinary team including hepatologists, transplant surgeons, endocrinologists, dietitians, radiologists, and other specialists when appropriate. CAP, controlled attenuation parameter; LT, liver transplantation; MASH, metabolic dysfunction-associated steatohepatitis; MASLD, metabolic dysfunction-associated steatotic liver disease; MDT, multidisciplinary team; MRE, magnetic resonance elastography; MRI-PDFF, magnetic resonance imaging-proton density fat fraction; VCTE, vibration-controlled transient elastography.

Within a risk-stratified monitoring framework (Figure 1), liver biopsy may serve as a subsequent assessment tool following a “trigger for escalation.” For example, liver biopsy may be considered in patients with suspected fibrosis progression, discordant non-invasive test results, or clinical suspicion of graft dysfunction, in order to clarify histopathological changes and guide subsequent management. Within this framework, patients may be broadly stratified according to metabolic risk and fibrosis status. Low-risk patients generally have minimal metabolic abnormalities and no clear evidence of steatosis or fibrosis. Intermediate-risk patients may present with one or more metabolic risk factors, post-transplant weight gain, mild steatosis, or possible early fibrosis. High-risk patients may include those with prior MASLD/MASH-related liver transplantation, multiple metabolic abnormalities, suspected fibrosis, or abnormal non-invasive test results. Low-risk patients may undergo routine metabolic assessment and periodic non-invasive follow-up, whereas moderate- to high-risk patients may require closer monitoring using VCTE and/or imaging-based evaluation. Escalation of assessment may be considered in patients with worsening metabolic abnormalities, progressive liver stiffness, increasing steatosis burden, discordant non-invasive test results, or suspected graft dysfunction. In selected high-risk patients, liver biopsy may be considered to further evaluate histological changes and guide subsequent management.

### 5.2 Limitations of serum-based non-invasive tests: challenges in LT recipients

In recent years, several serum-based non-invasive tests (NITs) have been widely applied for fibrosis risk assessment in MASLD, including the AST-to-platelet ratio index (APRI), fibrosis-4 index (FIB-4), and NAFLD fibrosis score (NFS) (43, 44). These scoring systems are derived from routine clinical and biochemical parameters and offer advantages such as simplicity, cost-effectiveness, and broad clinical accessibility, leading to their widespread use in the general MASLD population.

However, the applicability of these scores in LT recipients remains limited (45–49). Platelet counts may remain persistently low because of hypersplenism or residual portal hypertension, potentially leading to underestimation of fibrosis risk. In addition, aminotransferase levels may be influenced by immunosuppressive therapy, infection, or rejection episodes, thereby reducing the specificity of these scoring systems. Moreover, most currently available NITs were originally developed in patients with MASLD or chronic liver disease in the non-transplant setting, and their optimal cutoff values and predictive performance in LT recipients have not yet been adequately validated.

Accordingly, reliance on conventional serum-based non-invasive scores alone for the assessment of post-LT MASLD remains associated with considerable uncertainty. These tools should therefore be interpreted in conjunction with imaging-based evaluation and clinical risk stratification, rather than being used as standalone diagnostic modalities.

### 5.3 Non-invasive imaging assessment: from transient elastography to magnetic resonance-based techniques

During long-term follow-up of post-LT MASLD, conventional abdominal ultrasonography may be used for the initial assessment of hepatic steatosis; however, its sensitivity for detecting mild steatosis and early fibrosis remains limited, making it insufficient for dynamic longitudinal monitoring (50, 51). In parallel, serum-based non-invasive scores also show important limitations in LT recipients. Consequently, non-invasive elastography-based imaging techniques have increasingly emerged as a major approach for the longitudinal assessment of post-LT MASLD.

Among the available non-invasive elastography modalities, ultrasound-based vibration-controlled transient elastography (VCTE), combined with the controlled attenuation parameter (CAP), has become one of the most commonly used assessment tools in LT recipients (21, 52). VCTE primarily evaluates fibrosis severity through liver stiffness measurement, whereas CAP provides semiquantitative assessment of hepatic steatosis. Given its non-invasive nature, reproducibility, ease of use, and suitability for serial follow-up, FibroScan has been increasingly adopted as a first-line tool for dynamic monitoring of post-LT MASLD.

Nevertheless, FibroScan measurements may still be affected by factors including obesity, inflammation, and hepatic congestion (53–55). Although CAP has demonstrated value for steatosis screening, its ability to discriminate between different grades of steatosis remains limited, and diagnostic accuracy may decrease in patients with higher body mass index (BMI). Moreover, studies in LT recipients remain relatively limited, and standardized diagnostic cutoff values have not yet been fully established. Available evidence nevertheless suggests that CAP values of approximately 270–290 dB/m may provide reasonable diagnostic performance for hepatic steatosis (21, 56, 57).

On the other hand, although the diagnostic utility of VCTE for fibrosis assessment has been well validated in patients with MASLD, evidence in LT recipients remains comparatively limited. Current studies suggest that VCTE demonstrates good accuracy for identifying moderate-to-advanced fibrosis in LT recipients, with reported diagnostic cutoff values of approximately 10.5 kPa (57). Furthermore, previous meta-analyses have shown that VCTE provides superior overall diagnostic performance for non-invasive fibrosis assessment in LT recipients compared with serum-based indices such as APRI and FIB-4 (58).

Despite the favorable clinical accessibility of FibroScan, its diagnostic accuracy remains subject to certain limitations in specific patient populations. Consequently, magnetic resonance (MR)-based non-invasive assessment techniques have increasingly emerged as an area of growing interest. Magnetic resonance imaging–proton density fat fraction (MRI-PDFF) has demonstrated high accuracy for quantitative assessment of hepatic steatosis, whereas magnetic resonance elastography (MRE) has shown good diagnostic performance across different stages of fibrosis (59–62). In addition, several studies have suggested promising applications of MR-based techniques for steatosis assessment in LT recipients (63).

Compared with VCTE, MR-based techniques provide greater stability in patients with obesity and in more complex clinical settings. These modalities may be particularly useful for further evaluation in LT recipients with discordant non-invasive test results, suspected fibrosis progression, or multiple metabolic risk factors. However, due to their relatively high cost, prolonged examination time, and limited accessibility, MR-based techniques are currently used primarily for further assessment in high-risk patients or complex cases, and their widespread application in routine surveillance remains limited.

### 5.4 Risk-stratified dynamic monitoring strategies

Given the inherent limitations of individual diagnostic modalities, the management of post-LT MASLD should place greater emphasis on risk-stratified dynamic monitoring strategies rather than reliance on isolated test results. Based on currently available evidence and the specific characteristics of LT recipients, an integrated management pathway incorporating “baseline assessment–risk stratification–dynamic monitoring–trigger for escalation” may be established (Figure 1) (6, 12).

During the baseline assessment stage, initial risk stratification should integrate recipient metabolic status, prior history of MASLD, donor characteristics, immunosuppressive regimens, and cardiovascular risk factors. Among these, obesity, type 2 diabetes mellitus, dyslipidemia, hypertension, previous MASH-related cirrhosis, and persistent post-transplant weight gain are all associated with increased risks of disease recurrence or progression.

For low-risk patients, routine laboratory testing combined with abdominal ultrasonography may be sufficient for follow-up surveillance (Table 2). In contrast, patients at intermediate or high risk should undergo intensified non-invasive longitudinal monitoring, preferentially using VCTE combined with CAP for the assessment of steatosis and fibrosis, with follow-up evaluations performed every 6–12 months according to risk profile (21, 57). In patients with obesity, substantial metabolic risk burden, or discordant non-invasive test results, MRI-PDFF may be further applied for quantitative steatosis assessment, with magnetic resonance elastography (MRE) considered when additional fibrosis evaluation is required (59, 60, 62).

**Table 2:** Diagnostic tools and risk-stratified monitoring strategies for post-transplant MASLD.

Tool/modality	Primary purpose	Strengths	Limitations	Transplant-specific considerations	Recommended clinical role
Abdominal ultrasound	Initial screening for steatosis	Widely available, inexpensive, easy to perform	Limited sensitivity for mild steatosis and early fibrosis	Performance may be affected by obesity and operator dependency	Routine follow-up in low-risk patients
Non-invasive fibrosis scores (FIB-4)	Initial fibrosis risk assessment	Simple, low cost, easily accessible	Limited diagnostic accuracy in LT recipients	Platelet count and aminotransferase levels may be influenced by hypersplenism, immunosuppression, and infection	Adjunctive tool for risk stratification; not recommended as a standalone diagnostic method
VCTE (FibroScan)	Monitoring fibrosis progression dynamically	Non-invasive, repeatable, suitable for longitudinal follow-up	Results may be affected by obesity, inflammation, and hepatic congestion	Optimal cut-off values in LT recipients remain uncertain	First-line tool for dynamic longitudinal monitoring in moderate- to high-risk patients
CAP	Semi-quantitative assessment of hepatic steatosis	Can be performed simultaneously with VCTE	Limited ability to discriminate steatosis grades	Diagnostic accuracy may decrease in patients with high BMI	Screening and longitudinal monitoring of steatosis
MRI-PDFF	Quantitative assessment of liver fat content	High accuracy and reproducibility	High cost and limited accessibility	Studies in LT recipients remain limited	Advanced evaluation in obese patients or complex clinical scenarios
MRE	Accurate assessment of fibrosis	High diagnostic performance with less influence from obesity	Expensive and not widely available	LT-specific evidence remains limited	Used in high-risk patients or when fibrosis progression is suspected
Liver biopsy	Definitive diagnosis and histological staging	Gold standard for assessment of steatosis, inflammation, and fibrosis	Invasive with potential sampling error and complications	Not suitable for routine long-term surveillance	Decision-making tool following clinical escalation triggers

CAP, controlled attenuation parameter; LT, liver transplantation; MRE, magnetic resonance elastography; MRI-PDFF, magnetic resonance imaging–proton density fat fraction; VCTE, vibration-controlled transient elastography.

## 6 Risk-stratified personalized management

### 6.1 Integrated management paradigm: from liver disease to systemic risk control

The management of post-LT MASLD is more complex than that of MASLD in the non-transplant setting, as its development and progression are influenced by metabolic and transplant-specific factors (12, 17). Accordingly, care should focus on long-term integrated management strategy centered on comprehensive risk control (12, 38). Sex-specific hormonal factors may influence MASLD progression and support individualized management (64). Within this framework, cardiometabolic abnormalities, cardiovascular risk, renal dysfunction, and immunosuppression-related metabolic complications should all be incorporated into comprehensive assessment and intervention strategies (12, 17, 38). Within this framework, systematic control of cardiometabolic risk factors extends across multiple dimensions, including lifestyle intervention, optimization of immunosuppressive therapy, and metabolism-targeted treatment strategies (12, 17). Given that post-LT MASLD involves complex interactions among hepatology, transplant immunology, endocrinology, nutrition, and cardiovascular medicine, its management generally requires support from a multidisciplinary team (MDT) approach (65) (Figure 1). In practice, the MDT may include hepatologists, transplant surgeons, endocrinologists, dietitians, radiologists, and other specialists when appropriate.

### 6.2 Lifestyle intervention: a foundational and long-term core strategy

Lifestyle intervention represents the cornerstone of both prevention and treatment for post-LT MASLD and should be maintained throughout the entire disease trajectory (15, 17). Its primary goals are to achieve weight control, improve metabolic abnormalities, and reduce cardiovascular risk through dietary modification and regular physical activity (15, 66–69). Current management strategies increasingly emphasize a comprehensive approach characterized by “diet-first, exerci se-supported” intervention, in which dietary optimization serves as the central component and individualized exercise programs are incorporated to further improve metabolic status (17, 66–69).

With regard to dietary intervention, accumulating evidence supports the adoption of healthy dietary patterns like Mediterranean diet. This approach is characterized by high intake of unsaturated fatty acids, dietary fiber, and antioxidant-rich foods, together with reduced consumption of saturated fats and refined sugars, thereby contributing to improvements in insulin resistance, reduction of hepatic fat accumulation, and optimization of cardiometabolic health (15, 66–69). In addition, total caloric intake should be adjusted according to individual patient characteristics, with particular emphasis on alcohol avoidance to facilitate long-term weight management and metabolic improvement (17).

Compared with patients with MASLD in the non-transplant setting, exercise intervention in LT recipients requires a more individualized approach. During the early post-transplant period, rehabilitation should primarily focus on functional recovery and low-intensity activities such as walking. As recovery progresses, aerobic exercise and resistance training may be considered to improve cardiopulmonary fitness, sarcopenic obesity, and cardiometabolic risk profiles (66–69). Appropriate nutritional counseling and supervised exercise programs may further enhance long-term adherence and clinical benefit (15, 66–69).

Overall, lifestyle intervention is not only fundamental to the management of post-LT MASLD but also represents a central component of long-term metabolic risk control and overall prognostic improvement (15, 17). Sarcopenia is highly prevalent in end-stage liver disease and frequently persists or develops *de novo* after LT, with bidirectional relationships to metabolic syndrome and post-transplant MASLD. “Sarcopenic obesity” represents a particularly high-risk phenotype. The EASL nutrition guidelines (66) and supervised exercise studies (69) support integrated strategies combining nutritional optimization and structured exercise as core components for post-transplant MASLD prevention (70).

### 6.3 Optimization of immunosuppressive therapy: a transplant-specific therapeutic dimension

Optimization of immunosuppressive therapy represents a unique and critical component of post-LT MASLD management. The primary objective is to minimize metabolic adverse effects while maintaining graft stability (15, 21). Although the direct contribution of immunosuppressive agents to post-transplant steatosis has not been fully established, their metabolic side effects have been widely recognized and may indirectly contribute to disease progression through the promotion of insulin resistance, weight gain, and disturbances in glucose and lipid metabolism (6, 27).

Overall, current strategies increasingly favor short-term, low-dose corticosteroid regimens together with calcineurin inhibitor (CNI)-minimization approaches in order to reduce the risk of metabolic complications (15, 71). Among immunosuppressive agents, corticosteroids are considered one of the therapies most strongly associated with steatosis and metabolic abnormalities, whereas CNIs are also closely linked to disturbances in glucose and lipid metabolism (32). Because different immunosuppressive agents exhibit distinct metabolic profiles, treatment selection should be individualized according to each patient's metabolic risk burden.

Compared with CNIs, mammalian target of rapamycin (mTOR) inhibitors may offer certain advantages with respect to weight control, although their effects on lipid profiles appear less favorable (72). However, this potential advantage should not be interpreted as a general recommendation to use mTOR inhibitors in LT recipients with high metabolic risk. mTOR inhibitors may adversely affect lipid profiles and are associated with other transplant-specific considerations, including wound healing, proteinuria, renal function, and rejection risk (27, 72). Therefore, the current clinical approach is to individualize immunosuppressive regimens according to the patient's metabolic profile, graft stability, renal function, and overall risk-benefit balance, rather than selecting mTOR inhibitors solely for metabolic risk control (15, 27). However, the overall impact of mTOR inhibitors on post-LT MASLD remains controversial. A meta-analysis reported that only sirolimus use was associated with recurrent MASLD; however, the authors also noted that this finding may have been influenced by indication bias and other confounding factors, and therefore its true clinical significance requires further validation (6). In addition, metabolic differences also exist among individual CNIs. Tacrolimus appears to exert relatively less effect on hypercholesterolemia and hypertension, whereas cyclosporine A (CsA) may have a comparatively milder impact on glucose homeostasis (73).

Overall, individualized adjustment of immunosuppressive regimens based on metabolic risk stratification may help reduce long-term metabolic burden and improve long-term outcomes in patients with post-LT MASLD (15).

### 6.4 Emerging MASLD-related pharmacotherapies: potential benefits and practical challenges

In recent years, increasing attention has been directed toward emerging metabolic therapies for MASLD/MASH in the context of the growing burden of post-transplant metabolic dysfunction and obesity after LT, particularly glucagon-like peptide-1 receptor agonists (GLP-1RAs) and other novel therapeutic strategies. Given the high prevalence of post-transplant diabetes mellitus (PTDM) and obesity among LT recipients, the clinical use of these agents has progressively expanded in this population (74–77). Current management of PTDM generally follows a stepwise therapeutic approach, including lifestyle intervention, oral antidiabetic agents, and insulin therapy when necessary (78). Although dedicated studies in LT recipients remain limited for most antidiabetic medications, metformin, thiazolidinediones, and sulfonylureas have generally been considered safe in recipients of solid organ transplantation (SOT) (79–81).

GLP-1RAs have attracted substantial interest in the MASLD/MASH field because of their combined effects on weight reduction, improvement of insulin resistance, and reduction of hepatic fat accumulation. Emerging evidence suggests that these agents may not only improve glycemic control and promote weight loss, but also reduce hepatic steatosis and improve MASH-related histopathological features (82–85). In contrast, studies evaluating GLP-1RAs in LT recipients remain relatively limited. A study by Yakubu et al. demonstrated that treatment with GLP-1RAs (dulaglutide, liraglutide, and semaglutide) improved body weight and CAP values in LT recipients without apparent safety concerns or graft dysfunction (74). Similar findings have also been reported in several additional small-scale studies (75–77). Furthermore, a retrospective study showed that GLP-1RAs effectively improved glycemic control and reduced body weight in SOT recipients without significant adverse effects on tacrolimus levels, renal function, or transplant outcomes (86).

Overall, metabolic-targeted therapies such as GLP-1RAs may represent promising therapeutic options for post-LT MASLD. However, current evidence is still largely derived from small cohort studies and indirect evidence, and their long-term safety, potential drug interactions, and true clinical benefits in LT recipients require further validation. Accordingly, at the present stage, these agents may be more appropriately considered as components of individualized multidisciplinary management rather than routine first-line therapies for post-LT MASLD. The field of MASLD pharmacotherapy is rapidly evolving, with several novel agents showing promise, including thyroid hormone receptor-beta (THR-β) agonists (e.g., resmetirom), FGF21 analogs, and other hormone-based therapies (87, 88). THR-β agonists, in particular, have liver-targeted effects that reduce hepatic steatosis and inflammation. However, important considerations for the LT population include potential drug-drug interactions with immunosuppressive medications, long-term safety profiles, and the need for personalized approaches based on metabolic phenotype. Notably, clinical data specifically evaluating these novel agents in liver transplant recipients remain limited, highlighting an important area for future research.

### 6.5 Advanced metabolic interventions: bariatric surgery and emerging strategies

For LT recipients who achieve insufficient response to lifestyle and pharmacological interventions, advanced metabolic interventions such as bariatric surgery may be considered as adjunctive therapeutic options. Available evidence suggests that bariatric surgery may improve body weight, cardiometabolic risk factors, and hepatic steatosis. Among the available procedures, sleeve gastrectomy is generally regarded as the preferred approach because it may reduce the risk of impaired immunosuppressant absorption while preserving endoscopic access to the biliary tract (89).

However, prior abdominal surgery in LT recipients may result in intra-abdominal adhesions, thereby increasing the risk of perioperative complications and potentially limiting the feasibility of this strategy.

## 7 Conclusion

Post-LT MASLD is a highly heterogeneous metabolic disease driven by the complex interplay of recipient metabolic status, immunosuppressive therapy, and transplant-related factors. Current evidence suggests that the clinical implications of post-LT MASLD extend beyond hepatic steatosis alone, with fibrosis progression and systemic metabolic complications likely representing more important determinants of long-term prognosis.

Accordingly, future management strategies may need to shift from merely “detecting steatosis” toward multidimensional risk stratification and dynamic long-term management based on integrated clinical information. With continued advances in non-invasive assessment techniques, multimodal predictive models, and artificial intelligence-assisted risk evaluation, the management of post-LT MASLD is expected to progressively evolve toward a more precise and individualized risk-oriented paradigm.
